# Frequency of LTP (Pru p 3) and Profilin (Pru p 4) sensitisation in 1052 patients referenced to an immunoallergology department in Lisbon

**DOI:** 10.1186/2045-7022-4-S2-P54

**Published:** 2014-03-17

**Authors:** M Conceição Pereira Santos, Pedro Silva, Célia Costa, Letícia Pestana, Manuel Pereira Barbosa

**Affiliations:** 1 Faculdade Medicina Lisboa/Instituto Medicina Molecular, Lab. Imunologia Clinica, Lisboa, Portugal; 2 Hospital Santa Maria-Centro Hospitalar Lisboa Norte, Serviço de Imunoalergologia, Lisboa, Portugal

## Background

Lipid transfer proteins (LTP) and profilins are the most relevant panallergens in the clinical management of patients with allergy to pollen and plant food (mainly fresh fruits and tree nuts) in the Mediterranean countries. Knowledge of the prevalence of sensitization to these allergens in the population would be useful in clinical practice. In this study we analyze the frequency of LTP and profilin sensitization in patients referred to the outpatient clinic in Lisbon.

## Population and methods

1052 patients (64% female, median age 32 years) referred to the allergy department in Lisbon, during a period of 6 months, were submitted to an aeroallergens battery skin prick tests (SPT, Bial-Aristegui©, Spain) and to purified peach's LTP and profilin components (Pru p 3 and Pru p 4, respectively). In 26 sensitized patients, specific IgE (sIgE, UniCAP, Phadia Thermofisher©, USA) to these components was evaluated and a retrospective analysis of their clinical files was performed.

## Results

Of all tested patients, 375 (36%) were sensitized to at least one pollen (28.5% grass; 19.5% weed; 18.8% tree). In SPT we found 40(3.8%) Pru p 3 and 35 (3.3%) Pru p 4 sensitization. Co-sensitization was present in 7(0.7%) patients. Pollinosis was present in 70% of the LTP, 80% of profilin sensitized and 86% of the co-sensitized groups, whereas plant food allergy was present in 48%, 23% and 71% respectively [LTP: peach (15), plum (9) walnut (7), apple (3); profiling: melon (5), peach (3), apple (2)]. Cutaneous, respiratory and gastrointestinal manifestations of food allergy were significantly more frequent in the Pru p 3 and co-sensitized groups (p<.001). In 26 patients was found a correlation between SPT and sIgE positivity (p<0.01), but not between their wheal diameter and sIgE value (p=0.31). Wheal diameter (p=0.2) and sIgE value (p=0.24) were also not significantly higher in food-allergic patients.

## Conclusions

The frequency of LTP and profilin sensitization rate in our population was similar to those of other Mediterranean-based studies, when values are adjusted to the proportion of patients with pollen allergy. LTP sensitization, regardless of profilin co-sensitization, SPT wheal diameter or sIgE value was more often associated with plant-food allergy and presented more severe manifestations.

